# Addressing the Stigma of Mental Illness in Black Families and Communities in Ontario, Canada: Protocol for a Mixed Methods Study

**DOI:** 10.2196/66851

**Published:** 2025-05-09

**Authors:** Joseph Adu, Josephine P H Wong, Priscilla Boakye, Sebastian Gyamfi, Egbe B Etowa, Mark Fordjour Owusu

**Affiliations:** 1 Toronto Metropolitan University Toronto, ON Canada; 2 University of Windsor Windsor, ON Canada; 3 Higher Education Development Centre University of Otago Christchurch New Zealand

**Keywords:** racial discrimination, anti-Black racism, mental illness and stigma, Black families and communities, mental health, Ontario, study protocol, Canadian health sector, psychological wellbeing, Greater Toronto Area, social justice, Caribbean descent, African descent, depression, anxiety, stress, self-reported survey, Black individuals, community leader, empowerment

## Abstract

**Background:**

Racism and discrimination are among the factors perpetuating the persistent disparities within the Canadian health sector and related social and community services. Addressing issues of racism in Canada is crucial to reducing the mounting mental health disparities that subsequently impact the psychological well-being of diverse groups of people, particularly racialized and Black individuals. While some research has been conducted on mental illness–related stigma, very few peer-reviewed studies have attempted antistigma interventions to address mental health disparities in Black families and communities in Canada.

**Objective:**

This study aims to generate critical knowledge to reduce mental health disparities and mental illness stigma experienced by Black families and communities and engage them in cocreating a best-practice model to guide policy and programming. Our study intends to engage individuals living with or affected by mental illness, service providers, and community leaders in Black communities who are interested in stigma reduction activities and advocacy in Ontario, particularly in the Greater Toronto Area (GTA), including Durham and York Regions, London, Ontario, Brampton, and Ottawa.

**Methods:**

Informed by population health promotion approaches, critical race theory, and an intersectionality framework underpinned by social justice principles, this mixed methods study will engage individuals of Caribbean and African descent in 5 cities in Ontario. We will use online self-reported surveys with Black individuals (335/431) to assess depression, anxiety, stress, mental health knowledge, racial discrimination, and mental health stigma. We will also engage Black individuals (40/431) and service providers and community leaders (16/431) in focus groups and individual interviews (10/431). Results from the survey and focus groups will inform concept mapping activities with cross-sector leaders, decision makers, and community advocates (30/431) to cocreate a best-practice model to improve mental health outcomes in Black families and communities. Quantitative data will be analyzed using descriptive and inferential analyses through SPSS (IBM Corp). Qualitative data will be transcribed verbatim, and NVivo software (Lumivero) will be used for data management. We will apply Braun and Clarke’s framework of 6 phases in thematic analysis.

**Results:**

As of September 2024, the study has received ethical approval in Canada. We have completed data collection for phase one of the study and plans are far advanced to start recruitment for phases 2 and 3. Results from the study are expected in the last quarter of 2025 and the first quarter of 2026.

**Conclusions:**

This project will generate a novelty of knowledge to contribute to effective ways of addressing mental illness stigma and promoting mental health literacy in Black families and communities and other vulnerable populations. In addition, the knowledge gained from this study will be taken back to Black communities to empower affected individuals and their families.

**International Registered Report Identifier (IRRID):**

DERR1-10.2196/66851

## Introduction

Despite recent efforts in society to address social injustice and inequity, racism continues to violate Black Canadians’ human rights in every facet of society [1-4]. The mental health of Black families and communities continues to be negatively impacted by structural and systemic discrimination based on racism [2,3,5]. Racism and discrimination devalue and often discredit people of African descent [4,5], leading to trauma, frustration, and a sense of powerlessness, which adversely impact Black people’s mental health and quality of life [2,6-8]. Addressing issues of racism in Canada is crucial to reducing the mounting health disparities that subsequently impact the psychological well-being of diverse groups of people, particularly racialized and Black individuals [9-11].

Historical collective experiences of enslavement, displacement, and ongoing anti-Black racism produce and perpetuate intergenerational trauma and mental health problems among Black families and communities. Anti-Black racism has been explained as the systemic discrimination and prejudice embedded in institutions, policies, and practices targeted at people of African descent [12-14]. Evidence shows that anti-Black racism and racial discrimination are the causes of the persistent disparities within the Canadian health sector and related social and community services [15-17]. These disparities often result in poor access to both physical and mental health support among Black Canadians [11,18]. Related studies in Canada have found that the combined effect of racism and anti-Black racism experienced by Black people exposes them to emotional and psychological problems [1,5]. Mental illness stigma, a social construct that places shame on affected people, is another global health problem that appears to traumatize persons with mental illnesses or mental health challenges. For instance, a recent study by the Lancet Commission on ending stigma and discrimination in mental health underscored that stigma connected with mental illnesses is worse than the condition or diagnosis itself [19] and therefore has the tendency to prevent affected persons from accessing medical care and social support services [19-23]. Stigma and discrimination have various presentations that put affected persons and their families through emotional distress, which Thornicroft et al [19] termed double jeopardy. While mental illness stigma is a universal health problem, the stigma connected to a Black person diagnosed with mental health disorders in the Western world could be a threefold or triple-edged sword, as they have racism to deal with besides stigma and discrimination associated with mental illnesses. There is, however, a paucity of research on mental health and the stigma of mental illness experienced by Black families and communities in Canada, making it difficult to estimate the prevalence of mental health conditions [17,24].

The availability of socioeconomic resources is a social determinant of health as it allows individuals to improve the quality of their lives. Black people’s lack of equal access to these resources in Canada impacts their mental well-being and that of their families [16]. Barriers created by anti-Black racism in Canada are among the reasons behind the economic privileges enjoyed by White individuals to the disadvantage of Black people [12,14,25]. For instance, recent data from Canada showed that Black people of active working age have the highest unemployment rates (13.1% vs 7.7%) than nonracial minorities [12,26]. Likewise, it is documented in the 2016 Canadian Census that the unemployment rate of Black people of active working age was 10% when juxtaposed with the Canadian average of 6% for the same group with similar levels of education [27].

In addition, the unemployment rate among Black youth (15-24 years) was higher (24.3%) when contrasted with the Canadian youth average (15.5%) [28] and these gaps were more extensive during the COVID-19 pandemic [29,30]. Some ramifications of anti-Black racism are the high levels of poverty, homelessness, and substance use in Black communities [1,31], which in turn predisposes affected individuals to poor mental health. For example, the lack of formal employment due to structural and systemic racism can contribute to the rise in mental health problems in Black neighborhoods compared with other racialized and nonracialized communities [32,33]. Also, Black people in Canada are among the most uneducated due to their inability to break through the concealed systemic and structural barriers within various educational institutions [5,34]. The very few who have completed college and university are often greeted with frustrations and emotional distress given the hardship some endure throughout their education, and this can have a toll on their mental health. Furthermore, Black immigrants admitted into Canada through the economic immigration programs often find it difficult to secure employment that commensurate with their qualifications and occupational expertise. Underemployment over time exposes them to high levels of work-related stress as they are compelled to settle for mismatched jobs that deskill them [35-37].

Besides the socioeconomic issues faced by Black Canadians, the criminal justice system has been unjust to Black families and communities, which is connected to the remnants of slavery and colonialism [38]. Anti-Black racism has created systemic inequalities within the criminal justice system, where Black Canadians have experienced discrimination and prejudice more than any ethnic group when it comes to being survivors of crime and imprisonment [12,38]. For example, a recent review of the experiences of Black Canadians adds to existing evidence that the continued social discrimination confronting Black people is due to anti-Black racism and this has given rise to racial profiling and police brutality [5,39,40]. The overrepresentation of Black people within the criminal justice system resulting from unconscious bias and anti-Black racism eventually has ramifications for affected persons and their relatives’ psychosocial well-being. Arresting Black people and jailing them without a fair hearing could also lead to intergenerational trauma within Black families and communities. For instance, it is reported that Black persons are 20 times more likely than White persons to be shot dead by police in Toronto and this has created a sense of mistrust among Blacks in their dealings with the police [12]. Therefore, people of African and Caribbean origin continue to face these challenges produced by anti-Black racism and structural discrimination juxtaposed with White supremacy to produce or reproduce intergenerational distress that negatively affects the mental health of multiple generations of Black Canadians [11,24,41]. These incidents have become more egregious for Black refugees who have experienced premigration natural disasters or war-related traumas that have already impacted their mental health [2,42,43]*.* These premigration traumatic experiences, alongside postmigration experiences of anti-Black racism, not only compromise Black families and communities’ mental well-being but also impede their social integration and adaptation to achieve their life goals and aspirations [44].

The lack of sufficient data on Black families and communities mental health and mental illnesses is a barrier to inclusive mental health policies and strategic planning of accessible programs and services. In addition, early detection and treatment of mental illness result in good prognosis [45,46], and without inclusive research data, Black families and communities are further marginalized and unable to access mental health promotion resources and timely diagnosis and care. Given the documented evidence on the cycle of anti-Black racism and silence on mental illness stigma, mobilizing Black Canadians and communities to break the silence and promote critical dialogue on mental health literacy is essential to reduce the existing mental disparities and fill a scholarly gap.

### Study Objective

The overall goal of this proposed study is to generate critical knowledge to reduce mental health disparities and mental illness stigma experienced by Black families and communities and engage them in cocreating a best-practice model to guide policy and programming.

### Specific Objectives

- **Research objective (RO)1:** To identify and examine the sociocultural and structural factors that reproduce or produce and challenge community silence and stigma of mental illness in Black families and communities.
- **RO2:** To interrogate the role of racism and discrimination in perpetuating community silence and stigma of mental illness in Black families and communities.
- **RO3:** To assess the mental health literacy in Black families and communities and identify mental health facilitators at individual, family, and community levels.
- **RO4:** To engage Black families and communities in co-designing a best practices model to inform inclusive policy and culturally safe mental health programming.

### Methodology

#### Theoretical Approach

The project will be driven by a population health promotion framework underpinned by social justice principles. Guided by concepts of community empowerment and capacity building, the project will focus on creating supportive environments for health, strengthening local community action for health, and developing personal and collective skills in Black families and communities to enable them to take control of their mental health and well-being [47,48]. In addition, we will apply critical race theory [49-51] and an intersectionality framework [52,53] to interrogate how anti-Black racism operates to produce and perpetuate mental illness problems and related stigma at the individual, familial, and community levels. Further, we will investigate the prevailing mental health disparities in Black families and communities in Canada using the intersectionality framework.

#### Research Approaches

Mental illness-related stigma is a social construct often perpetrated by some family members and endorsed within varied social contexts [20,54]. While stigma experiences are everyday occurrences at all levels of society, interventions necessary to reduce mental illness stigma should involve multiple actors within communities to build collective capacities to fight all forms of stigma [54-56]. Therefore, this project will adhere to the principles and processes of community-based action research (CBAR). Community-based research methodology seeks to empower communities through research, education, and action [57-59]. In CBAR, community partners are actively involved in the research process compared with other conventional methodologies where community members or study participants are treated as passive participants [58]. A team of researchers well-versed in CBAR will collaborate meaningfully with identified Black community leaders, affected communities, and their representatives within the Greater Toronto Area (GTA) and other selected communities to set up project advisory committees to advise on research processes, community engagement, and ethical issues.

## Methods

### Overview

Given the complexity of the issues and the need for an in-depth understanding of the relationship between participants’ mental health literacy, psychosocial measures, and behavior change outcomes, a mixed-methods approach is considered appropriate for the proposed CBAR project. Specifically, an explanatory sequential design will be adopted for this project. In this design, we will collect and analyze quantitative data to inform our qualitative data collection to build on the initial results from the quantitative analysis. This approach is helpful to obtain additional information to explain critical quantitative results.

### Sample and Setting

The study will engage two populations: (1) individuals who self-identify as belonging to African, Caribbean, or Black communities, aged 18 years or older, living with or affected by mental illnesses, having family members living with mental illness, or having an interest in reducing mental illness stigma, living in the GTA, including Durham and York Regions, London, Ontario, Brampton, and Ottawa and (2) service providers and community leaders, aged 18 years or older, working with individuals and families of African, Caribbean, or Black communities and interested in reducing mental illness stigma in Black families and communities in the GTA, including Durham and York Regions, London, Ontario, Brampton, and Ottawa. Prospective participants will be reached using flyers, postcards, advertisements on social media platforms (including Facebook, Twitter, and LinkedIn), community collaborators’ e-newsletters, community service centers, family health practices, faith organizations, and educational institutions. Participants will also be recruited through word of mouth, personal contacts, and Black businesses (eg, barbershops, salons, restaurants, and grocery stores).

Data collection will start from October 2024 to August 2025. We will adhere to the approved study protocol to ensure that participants know their rights and confidentiality will be strictly ensured throughout all 3 phases of the study.

#### Phase One (RO#1-3)

An online survey will be used to assess participants’ mental health status using the Depression, Anxiety, and Stress Scale-21 Items (DASS-21) [60], stigma of mental illness using the Community Attitudes toward the Mentally Ill (CAMI) scale [61], discrimination using the Everyday Discrimination Scale (EDS) [62], and mental health literacy using the Mental Health Literacy Questionnaire-Short Version for Adults (MHLq-SVa) [63]. As an exploratory study, we will use a nonprobabilistic convenience sampling technique to recruit community members (n=335).

#### Phase Two (RO#1-3)

We will engage community members (n=40) and service providers/community leaders (n=16) in separate focus group discussions and 10 follow-up in-depth interviews. We will draw on the results of phase 1 to inform the interview guide to explore participants’ understanding of mental health and mental illness, the relationship between anti-Black racism and Black mental health, barriers, and facilitators in reducing mental illness stigma, contexts, and conditions to promote Black mental health. That is, survey results could play key roles in informing the interview guides for qualitative studies. Surveys provide diverse perspectives that allow the interviews to focus on areas critical to the study’s objective. Survey results often underscore critical themes and variables requiring additional inquiry through interviews. For example, qualitative interviews can focus on individuals to explore their unique lived experiences more extensively. In this way, the qualitative interviews help to critically examine a range of intricate issues within the survey population.

#### Phase Three (RO#4)

In this phase, we will apply an integrative knowledge translation approach to exchange knowledge and cocreate a best practices framework to address mental illness stigma and promote mental health in Black families and communities. We will engage a group of Black community members, service providers, cross-sector leaders, decision makers, and community advocates (n=30) in three concept mapping sessions to (1) share and discuss the results of phases 1 and 2; (2) engage every participant in brainstorming, sorting, and ranking essential elements of a best practices model to promote mental health equity in Black families and communities, use a computer program (GroupWidsom, Concept Systems Incorporated) to collate and generate concept maps based on everyone’s input; and (3) discuss different configuration of concept maps, decide on a model with priorities, and discuss follow-up action and strategies (Table 1).

**Table 1:** Study population and phase description.

Study phase	Target group	Involvement
Phase one (RO^a^#1-3): as an exploratory study, we will use a nonprobabilistic convenience sampling technique to recruit community members (n=335).	Individuals who self-identify as belonging to African, Caribbean, or Black communities, aged 18 years or older, living with or affected with mental illnesses, having family members living with mental illness, or having an interest in reducing mental illness stigma.	An online survey will be used to assess participants’ mental health status using the Depression, Anxiety and Stress Scale-21 Items (DASS-21) [60] (Lovibond and Lovibond, 1995), stigma of mental illness using the Community Attitudes toward the Mentally Ill (CAMI) scale [61], discrimination using the Everyday Discrimination Scale (EDS) [62], and Mental Health Literacy Questionnaire-Short Version for Adults (MHLq-SVa) [63].
Phase two (RO#1-3): engage community members (n=40) and service providers and community leaders (n=16) in separate FGDs^b^ and 10 follow-up IDIs^c^.	Service providers (psychiatrist, psychologist, social worker, and psychiatric nurse) and community leaders, aged 18 years or older, working with individuals and families of African, Caribbean, or Black communities, interested in reducing mental illness stigma in Black families and communities in the 5 communities.	Interview guides that explore participants’ understanding of mental health and mental illness, the relationship between anti-Black racism and Black mental health, barriers and facilitators in reducing mental illness stigma, and contexts and conditions to promote Black mental health.
Phase three (RO#4): engage a group of Black community members, service providers, cross-sector leaders, decision makers, and community advocates (n=30) in three concept mapping sessions.	Service providers, community leaders, and decision makers in the 5 selected cities who are committed to reducing racial discrimination, anti-Black racism, and mental illness stigma.	We will (1) share and discuss the results of phases 1 and 2, (2) engage every participant in brainstorming, sorting, and ranking essential elements of a best practices model to promote mental health equity in Black families and communities, (3) discuss different configurations of concept maps, decide on a model with priorities, and (4) discuss follow-up action and strategies.

^a^RO: research objective.

^b^FGD: focus group discussion.

^c^IDI: in-depth interview.

### Data Analyses Planning

Quantitative and qualitative data will be analyzed separately but interpreted together to deepen the understanding of the interaction between social factors and contexts of community silence on mental health and mental illness stigma. For the quantitative part, we will carry out varied data analyses using statistical software such as SPSS (IBM Corp) to achieve parts of objectives 1, 2, and 3. As preliminary steps, we will conduct univariate analysis to determine the distribution (frequencies and percentages) and measures of central tendencies (eg, mean, median, modes, etc) and dispersion (variance or SD) to capture distributions of sociodemographics (age, gender, relationship status, immigration status, places of birth, employment status, and educational attainment) and other scales (structural and mental health–related factors). As part of the preliminary data analysis process, we will conduct bivariate analysis to determine associations between sociodemographics and social and structural and mental health–related factors (eg, mental health, mental illness stigma, mental health literacy, and discrimination).

The preliminary data analysis process will provide insight into the variables’ selection for the final data analyses to achieve objectives 1-3. For objectives 1 and 2, we will use hierarchical linear regression modeling to determine the sociocultural and structural factors (including racism and discrimination) that produce or reproduce and challenge community stigma of mental illness in Black families and communities. Mental illness stigma will be the outcome variable measured by the CAMI scale [61], while sociocultural and structural factors (including racism and discrimination) will be included in the model as predictors. We will control the effect of sociodemographic factors in the model. Similarly, we will use hierarchical linear regression modeling to achieve part of objective 3, that is, to determine the factors associated with mental health in Black families and communities. The outcome variable (mental health) will be measured using DASS-21 [60], and predictor variables will include sociocultural factors, structural factors (including discrimination), and mental health literacy. Discrimination will be measured on the EDS [62] and the MHLq-SVa [63].

**Figure 1:**
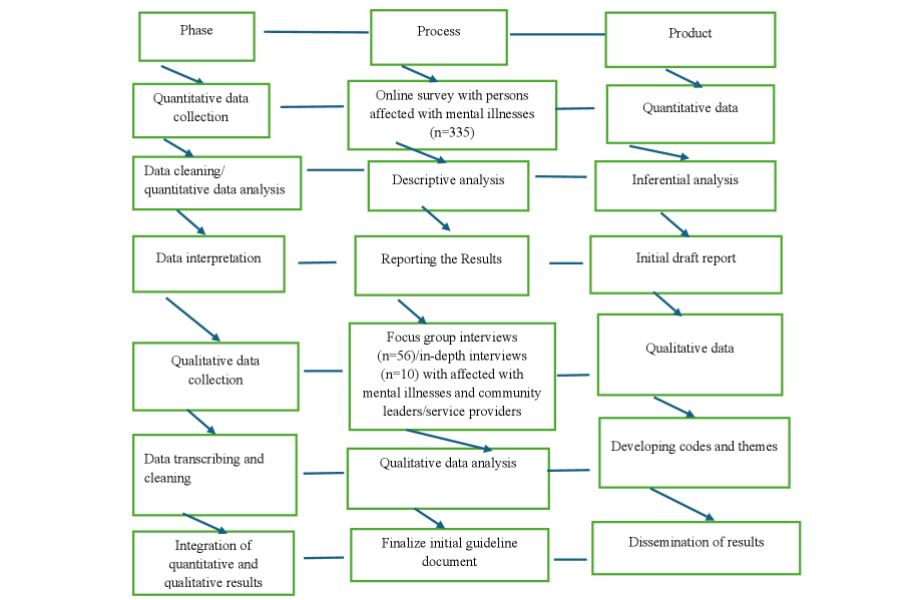
A sketch of the mixed methods study.

For the qualitative data, the voices of study participants informed by critical race and intersectionality theories will be inductively and deductively analyzed and interpreted using NVivo software (Lumivero) after transcription. Inductive thematic analysis, according to Nowell et al [64], offers stronger rigor and a methodical approach to produce meaningful and thought-provoking themes linked to the data without any preexisting coding frame or the investigator’s analytic presumptions. Other studies have also found that inductive analysis can be used in a versatile way within mixed-methods design to explore complex social issues [65,66]. The qualitative (focus group and in-depth interview data) analysis will follow Braun and Clarke’s [67,68] 6-stage framework for thematic analysis—familiarizing with the data, generating initial codes, searching for themes, reviewing the themes, defining the themes into a model, and writing the research report. Results from phase 1 will be used to engage participants in phase 2 discussions, and the findings from both phases will be integrated at the interpretation phase to inform integrative knowledge translation and concept mapping in phase 3 to cocreate the best practices model. A sketch of this study is shown in Figure 1.

### Ethics Approval

The study protocol has been approved by the Research Ethics Board of Toronto Metropolitan University (REB 2024-040).

## Results

As of September 2024, the study has received approval in Canada. We have completed data collection for phases 1 and 2 of the study. That is, as of April 2025, the online survey with persons affected with mental illnesses and related stigma (n=335), four focus group discussions with persons affected with mental illnesses and related stigma (n=37), and two group interviews with community leaders and community service providers (n=19) have been completed. All audio recordings have been transcribed and preliminary data analysis is currently ongoing.

Plans are far advanced to start recruitment for phases 3 and 4. Our online survey started in October and ended in December 2024. Data collection for this study ends on July 08, 2025. Results of the study are expected in the last quarter of 2025 and the first quarter of 2026.

## Discussion

### Overview

This study with Black families and communities aims to generate critical knowledge to reduce mental health disparities, mental illness stigma, and anti-Black racism experienced by Black families and communities, while engaging Black families and communities in cocreating a best practices model to guide policy and programming. The results obtained from the three data sources (ie, quantitative, qualitative, and concept mapping) will form the basis for cocreating a best practices model to address mental illness–related stigma and promote mental health in Black families and communities. This project will be among the first few studies exploring the contextual needs of Black people to address mental health challenges in Black families and communities. While several studies have examined mental health and mental illness–related stigma among Black people in Canada [17,34,69], very few have attempted to co-design a contextual framework to deal with the growing mental health disparities in Black communities [70,71]. We will compare and contrast our findings with other related studies in different jurisdictions in our quest to cocreate a best-practices model that bridges the existing mental health disparity gap between Black and White communities in Canada. The involvement of community leaders and service providers in this study will inspire participants and community members to eagerly endorse and promote interventions and social support networks necessary to sustain the mental well-being of Black families and communities.

### Dissemination of Findings

The findings from study will be shared with our community partners, who will disseminate the results through their local and national networks, as well as at Black community events and activities, after all results have been reviewed and approved by both academic and community research advisory committees. The findings will also be disseminated via academic conferences, peer-reviewed publications, and online open-access papers (eg, The Conversation). We will engage with the local media to raise awareness of the impact of anti-Black racism on the mental health of Black families and communities based on our findings. In addition, we will generate infographics and distribute them to our research team to share on their social media handles for a wider scope. The infographics will also be shared with policy makers, and further discussions on the study results will be arranged to inform policy decisions. Furthermore, we will share a summary of the results with all participants who requested copies during the project recruitment phase.

### Strengths and Limitations

The study’s strengths include the use of mixed methods, which could considerably enhance the depth and reliability of our study by combining both qualitative and quantitative strategies. The integrative analytic approach in mixed methods allowed us to address critical research questions more extensively than using a single method. The project team comprised both quantitative and qualitative experts, which paved the way for the exchange of knowledge through mentorship. However, there were some limitations to the project such as the lack of funds and other resources to enroll more people in the study and the inability to monitor the online survey participants. In addition, the use of two approaches for data collection and analysis proved to be time-consuming.

### Conclusion

This project will generate a novelty of knowledge that will contribute to effective ways of addressing the stigma of mental illness and promoting mental health literacy in Black families and communities and other vulnerable populations. Also, the new knowledge gained from this study will be taken back to the Black communities to empower affected individuals and their families to promote the needed interventions. We anticipate that the best-practice model cocreated by the project will contribute to research innovation in Canada and beyond.

## Abbreviations

CAMI: Community Attitudes toward the Mentally Ill
CBAR: community-based action research
DASS-21: Depression, Anxiety and Stress Scale-21 Items
EDS: Everyday Discrimination Scale
GTA: Greater Toronto Area
MHLq-SVa: Mental Health Literacy Questionnaire-Short Version for Adults
RO: research objective

## References

[1] Government of Canada (2023). Discussion paper on systemic racism. Canadian Human Rights Commission.

[2] King DD, Fattoracci ESM, Hollingsworth DW, Stahr E, Nelson M (2023). When thriving requires effortful surviving: delineating manifestations and resource expenditure outcomes of microaggressions for Black employees. J Appl Psychol.

[3] Mooten N (2021). Racism, discrimination and migrant workers in Canada: evidence from the literature. Government of Canada.

[4] Sue D, Capodilupo C, Torino G, Bucceri J, Holder A, Nadal K, Esquilin M (2007). Racial microaggressions in everyday life: implications for clinical practice. Am Psychol.

[5] Williams MT, Khanna Roy A, MacIntyre MP, Faber S (2022). The traumatizing impact of racism in Canadians of colour. Curr Trauma Rep.

[6] Hicken MT, Lee H, Ailshire J, Burgard SA, Williams DR (2013). "Every shut eye, ain't sleep": the role of racism-related vigilance in racial/ethnic disparities in sleep difficulty. Race Soc Probl.

[7] Pitcan M, Park‐Taylor J, Hayslett J (2018). Black men and racial microaggressions at work. Career Dev Q.

[8] Williams MT (2020). Microaggressions: clarification, evidence, and impact. Perspect Psychol Sci.

[9] (2018). Let's talk: racism and health equity. National Collaborating Centre for Determinants of Health.

[10] Gajaria A, Guzder J, Rasasingham R (2021). What's race got to do with it? A proposed framework to address racism's impacts on child and adolescent mental health in Canada. J Can Acad Child Adolesc Psychiatry.

[11] (2018). Public Health Agency of Canada. Key health inequalities in Canada: a national portrait.

[12] DasGupta N, Shandal V, Shadd D, Segal A (2020). The pervasive reality of anti-Black racism in Canada.

[13] Kumsa MK, Mfoafo-M'Carthy M, Oba F, Gaasim S (2014). The contours of anti-black racism: engaging anti-oppression from embodied spaces. J Crit Anti-Oppressive Soc Inq.

[14] Oyeniran C (2022). Anti-Black racism in Canada. The Canadian Encyclopedia.

[15] Phillips-Beck W, Eni R, Lavoie JG, Avery Kinew K, Kyoon Achan G, Katz A (2020). Confronting racism within the Canadian healthcare system: systemic exclusion of first nations from quality and consistent care. Int J Environ Res Public Health.

[16] Social determinants and inequities in health for Black Canadians: a snapshot. Government of Canada.

[17] Salami B, Idi Y, Anyieth Y, Cyuzuzo L, Denga B, Alaazi D, Okeke-Ihejirika P (2022). Factors that contribute to the mental health of Black youth. CMAJ.

[18] Olanlesi-Aliu A, Alaazi D, Salami B (2023). Black health in Canada: protocol for a scoping review. JMIR Res Protoc.

[19] Thornicroft G, Sunkel C, Alikhon Aliev A, Baker S, Brohan E, El Chammay R, Davies K, Demissie M, Duncan J, Fekadu W, Gronholm PC, Guerrero Z, Gurung D, Habtamu K, Hanlon C, et al (2022). The lancet commission on ending stigma and discrimination in mental health. Lancet.

[20] Adu J, Oudshoorn A, Anderson K, Marshall CA, Stuart H (2023). Experiences of familial stigma among individuals living with mental illnesses: a meta-synthesis of qualitative literature from high-income countries. J Psychiatr Ment Health Nurs.

[21] Corrigan PW, Mittal D, Reaves CM, Haynes TF, Han X, Morris S, Sullivan G (2014). Mental health stigma and primary health care decisions. Psychiatry Res.

[22] (2020). A primer to reduce substance use stigma in the Canadian Health System. Government of Canada.

[23] (2019). Special initiative for mental health (2019-2023). World Health Organization.

[24] Cénat JM, Kogan C, Noorishad PG, Hajizadeh S, Dalexis RD, Ndengeyingoma A, Guerrier M (2021). Prevalence and correlates of depression among Black individuals in Canada: the major role of everyday racial discrimination. Depress Anxiety.

[25] Do D (2020). Statistics Canada. Canada's Black population: education, labour and resilience.

[26] (2021). Study: a labour market snapshot of Black Canadians during the pandemic. Statistics Canada.

[27] Houle R (2020). Statistics Canada. Changes in the socioeconomic situation of Canada's Black population, 2001 to 2016.

[28] Spiteri S (2023). What can the data tell us about Black Canadians and the labour market?. Labour Market Information Council (LMIC).

[29] Ng ES, Gagnon S (2020). Employment gaps and underemployment for racialized groups and immigrants in Canada: current findings and future directions.

[30] (2021). CHPO Sunday edition: the impact of COVID-19 on racialized communities. Public Health Agency of Canada.

[31] (2022). Inequalities in health of racialized adults in Canada. Public Health Agency of Canada.

[32] Banaji MR, Fiske ST, Massey DS (2021). Systemic racism: individuals and interactions, institutions and society. Cogn Res Princ Implic.

[33] (2022). Understanding systems: the 2021 report of the National Advisory Council on poverty. Government of Canada.

[34] Fante-Coleman T, Jackson-Best F (2020). Barriers and facilitators to accessing mental healthcare in Canada for Black youth: a scoping review. Adolescent Res Rev.

[35] Fung KPL, Liu JJW, Sin R, Bender A, Shakya Y, Butt N, Wong JPH (2022). Exploring mental illness stigma among Asian men mobilized to become community mental health ambassadors in Toronto Canada. Ethn Health.

[36] Lauber C, Rössler W (2007). Stigma towards people with mental illness in developing countries in Asia. Int Rev Psychiatry.

[37] Virgolino A, Heitor MJ, Carreiras J, Lopes E, Øverland S, Torp S, Guðmundsdóttir D, Miguel JP, Fátima Reis M, Santos O (2017). Facing unemployment: study protocol for the implementation and evaluation of a community-based intervention for psychological well-being promotion. BMC Psychiatry.

[38] (2023). Engaging with Black communities to address systemic discrimination and overrepresentation in the criminal justice system. Government of Canada.

[39] Cotter A (2022). Perceptions of and experiences with police and the justice system among the Black and Indigenous populations in Canada. Statistics Canada.

[40] Wortley S, Jung M (2020). Racial disparity in arrests and charges: an analysis of arrest and charge data from the Toronto Police Service. Ontario Human Rights Commission.

[41] Giwa S, Mullings DV, Adjei PB, Karki KK (2020). Racial Erasure: the silence of social work on police racial profiling in Canada. J Hum Rights Soc Work.

[42] Vukčević Marković M, Bobić A, Živanović M (2023). The effects of traumatic experiences during transit and pushback on the mental health of refugees, asylum seekers, and migrants. Eur J Psychotraumatol.

[43] (2021). Mental health and forced displacement. World Health Organization.

[44] Donato KM, Ferris E (2020). Refugee integration in Canada, Europe, and the United States: perspectives from research. Annals Am Acad Pol & Soc Sci.

[45] Colizzi M, Lasalvia A, Ruggeri M (2020). Prevention and early intervention in youth mental health: is it time for a multidisciplinary and trans-diagnostic model for care?. Int J Ment Health Syst.

[46] Lawrie SM, Fletcher-Watson S, Whalley HC, McIntosh AM (2019). Predicting major mental illness: ethical and practical considerations. BJPsych Open.

[47] Hamilton N, Bhatti T (1996). Population health promotion: an integrated model of population health and health promotion. Public Health Agency of Canada.

[48] World Health Organization (1986). Ottawa Charter for Health Promotion. Health Promot Int.

[49] Daftary A (2018). Critical race theory: an effective framework for social work research. J Ethn Cult Diversit.

[50] Ford CL, Airhihenbuwa CO (2010). Critical race theory, race equity, and public health: toward antiracism praxis. Am J Public Health.

[51] Graham L, Brown-Jeffy S, Aronson R, Stephens C (2011). Critical race theory as theoretical framework and analysis tool for population health research. Critical Public Health.

[52] Collins PH, Bilge S (2016). Intersectionality ? Key Concepts. Intersectionality ? Key Concepts.

[53] Collins PH, Bilge S (2020). Intersectionality.

[54] Guruge S, Fung KPL, Sidani S, Este D, Morrow M, McKenzie K, Wong JPH (2018). Study protocol: mobilizing asian men in Canada to reduce stigma of mental illness. Contemp Clin Trials.

[55] Corbiere M, Samson E, Villotti P, Pelletier JF (2012). Strategies to fight stigma toward people with mental disorders: perspectives from different stakeholders. The Scientific World Journal.

[56] Li A, Wong JP, Fung K, Luyombia H, Bisignano C, Maitland D, Zurowski M (2015). Community champions HIV/AIDS advocates mobilization project: Reducing stigma and advancing equity through collective action.

[57] Gullion JS, Tilton A (2020). Researching with: a decolonizing approach to community-based action research. Brill. CJAR.

[58] Minkler M, Wallerstein N (2010). Community-Based Participatory Research for Health: From Process to Outcomes.

[59] Israel BA, Schulz AJ, Parker EA, Becker AB (1998). Review of community-based research: assessing partnership approaches to improve public health. Annu Rev Public Health.

[60] Lovibond PF, Lovibond SH (1995). The structure of negative emotional states: comparison of the Depression Anxiety Stress Scales (DASS) with the Beck Depression and Anxiety Inventories. Behav Res Ther.

[61] Taylor SM, Dear MJ (1981). Scaling community attitudes toward the mentally ill. Schizophr Bull.

[62] Williams DR, Yan YU, Jackson JS, Anderson NB (1997). Racial differences in physical and mental healthocio-economic status, stress and discrimination. J Health Psychol.

[63] Campos L, Dias P, Costa M, Rabin L, Miles R, Lestari S, Feraihan R, Pant N, Sriwichai N, Boonchieng W, Yu L (2022). Mental health literacy questionnaire-short version for adults (MHLq-SVa): validation study in China, India, Indonesia, Portugal, Thailand, and the United States. BMC Psychiatry.

[64] Nowell LS, Norris JM, White DE, Moules NJ (2017). Thematic analysis: striving to meet the trustworthiness criteria. Int J Qual Methods.

[65] Creswell JW, Clark VL (2017). Designing and Conducting Mixed Methods Research.

[66] Proudfoot K (2022). Inductive/deductive hybrid thematic analysis in mixed methods research. J Mix Methods Res.

[67] Braun V, Clarke V, Cooper H, Camic PM, Long DL, Panter AT, Rindskopf, D, Sher KJ (2012). APA Handbook of Research Methods in Psychology, Vol. 2. Research Designs: Quantitative, Qualitative, Neuropsychological, and Biological.

[68] Braun V, Clarke V, Sage Caplan, S, Paris, M, Whittemore R, Desai M, Dixon J, Alvidrez J, Escobar, J, Scahill L (2022). Conceptual and Design Thinking for Thematic Analysis. Qualitative Psychology.

[69] Cénat JM, Dalexis RD, Darius WP, Kogan CS, Guerrier M (2023). Prevalence of current PTSD symptoms among a sample of Black individuals aged 15 to 40 in Canada: the major role of everyday racial discrimination, racial microaggresions, and internalized racism. Can J Psychiatry.

[70] (2022). Dismantling anti-Black racism. Centre for Addiction and Mental Health.

[71] Varcoe C, Browne A, Perrin N, Wilson E, Bungay V, Byres D, Wathen N, Stones C, Liao C, Price ER (2022). EQUIP emergency: can interventions to reduce racism, discrimination and stigma in EDs improve outcomes?. BMC Health Serv Res.

